# Enterotype-specific microbial biomarkers of immune checkpoint inhibitor response revealed by large-scale integrated metagenomic analysis

**DOI:** 10.1007/s00262-026-04432-w

**Published:** 2026-05-26

**Authors:** Francesco Candeliere, Enrico Busi, Sara Cerri, Laura Sola, Matteo Lombardi, Stefano Greco, Sara Pedroni, Alberto Amaretti, Stefano Raimondi, Chiara Chiavelli, Maria Giuseppa Vitale, Federica Bertolini, Roberta Depenni, Giorgia Franchini, Massimo Dominici, Maddalena Rossi

**Affiliations:** 1 Department of Life Sciences, University of Modena and Reggio Emilia, Via Campi 103, 41125 Modena, Italy; 2 Operative Unit of Oncology, Department of Oncology, ASST Cremona Hospital of Cremona, 26100 Cremona, Italy; 3 Department of Physics, Informatics and Mathematics, University of Modena and Reggio Emilia, Modena, Italy; 4 Division of Oncology, Department of Medical and Surgical Sciences for Children and Adults, University of Modena and Reggio Emilia, Largo del Pozzo 71, Modena, Italy; 5 Division of Oncology, Department of Oncology and Hematology, University of Modena and Reggio Emilia and University Hospital of Modena, Modena, Italy; 6 Biogest-Siteia, University of Modena and Reggio Emilia, Modena, Italy

**Keywords:** Gut microbiota, Metagenomics, Immunotherapy, Enterotype, Machine learning

## Abstract

The gut microbiota appears to play a critical role in modulating antitumor immune responses and influencing the efficacy of cancer immunotherapy drugs such as immune checkpoint inhibitors. However, the identification of consistent microbial biomarkers of response remains a significant challenge. This lack of consensus is largely driven by multi-source heterogeneity, including geographic variations in lifestyle, and high inter-individual variability. We hypothesize that these inconsistencies arise because microbiome composition is not uniform but organized into distinct enterotypes. To address this, we performed an integrated metagenomic analysis of 569 fecal samples from oncological patients affected by different tumor types treated with immunotherapy. The samples were clustered into two main enterotypes, E1 and E2, each of them containing two subclusters. A total of 166 species (e.g., *Collinsella* spp., *Blautia* spp., *Bacteroides* spp.) were identified as enterotype-specific biomarkers. A preliminary independent concordance assessment of these biomarkers was conducted in 19 oncologic patients with exceptional response to immunotherapy, providing an initial confirmation of selected enterotype-associated signals. Furthermore, we evaluated the predictive potential of gut microbiota profiles for immunotherapy outcomes through machine learning techniques. The models showed encouraging, albeit moderate, performance in the heterogeneous full dataset, supporting the potential of microbiome-based stratification as an exploratory framework for patient classification, while indicating that further validation is needed before clinical application.

**Supplementary Information:**

The online version contains supplementary material available at 10.1007/s00262-026-04432-w.

## Introduction

Cancer immunotherapy is a treatment approach that fights cancer by selectively regulating and enhancing the activity of the immune system. In recent years, the introduction of cancer immunotherapeutic approaches, such as immune checkpoint inhibitors (ICIs) in clinical practice, have profoundly reshaped the treatment landscape of several solid tumors, including malignant melanoma (MM), non-small cell lung cancer (NSCLC), and renal cell carcinoma (RCC) [1–3]. Although ICIs (like anti-PD-1, anti-PD-L1 and anti-CTLA-4 antibodies) have led to durable and sometimes remarkable clinical responses in a subset of patients, a substantial proportion fails to benefit from these therapies due to the development of primary or acquired resistance. Elucidating novel ICI predictive biomarkers underlying response and non-response to immunotherapy represents a major clinical and scientific challenge.

Growing evidence indicates that gut microbiota plays a critical role in modulating antitumor immune responses and influencing the efficacy of immune checkpoint blockade [4, 5]. Several landmark studies have identified distinct gut microbial taxa as putative biomarkers of response to immune checkpoint inhibitors, highlighting the enrichment of *Akkermansia muciniphila*, *Faecalibacterium prausnitzii*, specific *Bacteroides* species (e.g., *B. caccae*, *B. ovatus*), and members of the genera *Blautia* and *Collinsella* in patients achieving favorable clinical outcomes [6–12]. These associations, observed across MM, NSCLC, and RCC cohorts, suggest that specific compositional and functional configurations of the gut microbiome may enhance systemic antitumor immunity and modulate responsiveness to antiPD-1/PD-L1 or antiCTLA-4 drugs.

However, the identification of consistent and universal microbial biomarkers of response has proven difficult. The absence of a clear consensus across studies can be attributed to multiple sources of heterogeneity. Variations in sample handling procedures and DNA isolation methods may introduce technical biases that affect downstream analyses. Differences in dietary habits and patterns of medication across geographic regions further contribute to inconsistencies in microbial profiles. Limited sample sizes and insufficient statistical power can also hinder the detection of reproducible associations. In addition, responder groups often display substantial inter-individual variability in their microbial composition. Functional similarities may be present, yet the specific taxa driving these effects can differ between cohorts. Broader cohort-related influences, including demographic features and analytical workflows, remain a significant challenge in microbiome research.

One possible explanation for this inconsistency is that microbiome composition is not uniform across individuals but can be broadly organized into distinct compositional configurations, commonly referred to as enterotypes. Failure to account for this underlying microbiome stratification may obscure response-associated signals when analyzing heterogeneous cohorts. Stratifying patients into microbiome-defined groups could therefore reduce biological noise and enable the identification of response-related biomarkers that are specific to particular microbial contexts rather than universally shared.

In this study, we performed an integrated metagenomic analysis of a large collection of 569 fecal samples from oncological patients treated with immune checkpoint inhibitors, encompassing multiple tumor types. Our objectives were to: (i) investigate the relationship between gut microbiome composition and clinical response to immunotherapy; (ii) define and preliminarily assess enterotype-specific microbial biomarkers associated with therapeutic response; and (iii) evaluate the predictive potential of microbiome profiles using machine learning approaches. By combining large-scale metagenomic profiling, enterotype-based stratification, preliminary biomarker assessment, and artificial intelligence-driven classification, this work aims to provide a proof-of-concept framework for microbiome-informed stratification and biomarker discovery in heterogeneous cohorts.

## Materials and methods

### Dataset definition

The dataset utilized in this study included fecal samples metagenomes retrieved from 9 studies, downloaded from the public database NCBI SRA. The dataset was assembled with metagenomes from patients affected by MM, NSCLC and RCC. Only metagenomes from responders (R) and non-responders (NR) were included in the dataset, while stable patients were excluded. Patient response was retrieved from Bioprojects metadata and was assessed by the respective original authors according to the response evaluation criteria in solid tumors 1.1 (RECIST 1.1). As R, we selected those corresponding to the criteria “Complete Response (CR)” and “Partial Response (PR)”, while as NR those labeled as “Progressive disease (PD)” and “Dead (D)”. Those marked as “Stable disease (SD)” were considered as stable and discarded from the analysis. Overall, the analyzed dataset included 569 samples. The Bioproject identifiers of the utilized studies are reported in Table 1 and the samples accession number are available in Suppl. Spreadsheet 1.

**Table 1:** List of the bioprojects utilized to retrieve the metagenomes included in the dataset utilized in this study. number of R and NR subjects, type of tumor and country of provenance are reported

Bioproject	N° R/N° NR	Tumor	Country	Reference
PRJEB43119	64/71	Melanoma	UK, Netherland, Spain	Lee et al., 2022 [10]
PRJNA866654	8/4	Non-small cell Lung Cancer	North America	Liu et al., 2022 [27]
PRJEB22863	26/56	Renal cell carcinoma	France	Routy et al., 2017 [7]
PRJNA751792	70/113	Non-small cell lung cancer	France	Derosa et al., 2022 [11]
PRJNA672867	33/14	Melanoma	North America	Davar et al., 2021 [28]
PRJNA399742	15/12	Melanoma	North America	Matson et al., 2018 [8]
PRJNA397906	19/15	Melanoma	North America	Frankel et al., 2017 [6]
PRJNA762360	15/7	Melanoma	North America	McCulloch et al., 2022 [12]
PRJNA541981	12/15	Melanoma	North America	Peters et al., 2019 [9]

## Raw reads preprocessing and metagenomes profiling

Raw reads were downloaded as FASTQ files through SRA Toolkit v 3.1.1 (github.com/ncbi/sra-tools). Quality check and primer presence were assessed utilizing FastQC v0.11.8 [13], in order to assure that only high-quality reads (length > 50 bp; quality score > 20) were further analyzed. When necessary, the tool Trimmomatic v0.40 [14] was used for primer removal, with ILLUMINACLIP default setting. Reads originating from human contamination were removed through the mapping of the human genome assembly GRCh38 with the tool bowtie2 [15], a tool specialized to map short reads on reference sequences.

The microbial composition of each sample was evaluated using Kraken 2 [16] with the Unified Human Gastrointestinal Genome database v2.0.1 (UHGG) [17]. Abundances were estimated at species level using Bracken [18]. All analyses were performed with default parameters for k-mer length, minimizer length, and minimizer spacing. Reports produced by Bracken were converted to *biom* files through kraken-biom [19] and imported into QIIME2 [20] for alpha- and beta-diversity analysis. Alpha diversity was evaluated with Chao1, Pielou, and Shannon indices, and Kruskal–Wallis test was applied to assess statistical significance. Beta diversity was investigated by calculating Bray–Curtis dissimilarity on the Bracken-derived relative abundance profiles, and the resulting distance matrix was analyzed through Principal Coordinates Analysis (PCoA). PERMANOVA was used to determine statistical significance in the differences between R and NR.

## Clustering and biomarkers identification

Metagenomes clustering was assessed using the partitioning around medoid (PAM) algorithm through the function *pamk* of the R package *fpc* [21]. Cluster attribution of each sample was determined using the Bray–Curtis distance matrix computed from species-level relative abundance profiles generated by Bracken, normalized at 100%. The process was repeated iteratively to identify smaller clusters and refine the clustering structure.

LEfSe (Linear discriminant analysis Effect Size) [22] analysis was conducted to identify biomarkers characterizing R and NR samples in the complete dataset and in each cluster.

The presence of differences in the distribution of R and NR in the clusters and subclusters, taking into account also the diverse tumors, was evaluated with Chi-squared and Fisher’s exact test. Both tests were carried out with R v.4.4.2 with the functions *chisq.test* and *fisher.test*, respectively.

## Assessment of the consistency of responder-associated biomarkers

A preliminary independent concordance assessment of the responder-associated biomarkers identified by LEfSe was conducted with 19 metagenomes from patients with MM, NSCLC or RCC treated with first-line immunotherapy at Modena Cancer Center, Italy (Modena Oncology Cohort—MOC; Bioproject PRJNA1421997; Suppl. Spreadsheet 1; Suppl. Table 1), from 2016 to 2022. Patients were enrolled on the basis of exceptional clinical benefit after ICI (anti-PD-1 or anti-PD-1 + anti-CTLA-4; treatments in association with chemotherapy or small molecules were excluded). Specifically, patients with a CR or PR lasting more than 2 years were selected [23, 24]. This cohort was not designed to provide a balanced predictive validation against local non-responder controls, but rather to verify whether selected responder-associated signals could also be observed in an independent group of exceptional responders. The recruitment was conducted in accordance with the protocol approved by the local research ethics committee (reference number 720/2023/TESS/AOUMO, Comitato Etico Area Vasta Emilia Nord, Azienda Ospedaliero-Universitaria di Modena, Italy) and the subjects provided informed consent. The raw reads were subjected to the same preprocessing previously described, and their composition profile was calculated with the same pipeline, utilizing Kraken2 and Bracken with UHGG database. Each MOC sample was attributed to a cluster and subcluster considering the Bray–Curtis dissimilarity between the sample and the medoid sample of each cluster and subcluster. The abundances of biomarkers identified for each cluster/subcluster were then examined in the MOC samples, and the probability of belonging to the R or NR distribution derived from the whole dataset was calculated with a bootstrap confidence score with R v4.4.2 and the *boot* package v1.3-v31 [25]. The score was based on 1000 iterations where a standardized distance was calculated as the difference between the abundance value in an MOC responder sample and the median of the R/NR distribution, divided by its interquartile range (IQR = Q3—Q1). A biomarker was considered supported in this preliminary concordance assessment when it was predicted in the R distribution in 80% of the MOC samples attributed to that cluster/subcluster with a bootstrap value > 0.9. For enterotypes for which only 2 to 5 assessment samples were available, the biomarker was confirmed when all abundances in the available samples matched the R distribution (100%).

## Machine learning analysis

The whole dataset (WD) of fecal microbiota from 569 oncological patients was analyzed using machine learning methods to perform classification tasks aimed at distinguishing responders (CR + PR) from non-responders (PD + D) to immunotherapy treatment. The microbial composition of the dataset was examined both at the species and at the genus level, to evaluate the impact of taxonomic resolution and the description of the microbial community on classification performance. The MOC was used as an external responder-only exploratory test set, and results from this cohort were interpreted as concordance of responder assignment rather than as a full predictive validation.

Machine learning analysis was preceded by preprocessing methods for data reduction and normalization. For data reduction, Principal Component Analysis (PCA), Kernel Principal Component Analysis (KPCA), t-distributed Stochastic Neighbor Embedding (t-SNE), Principal Coordinates Analysis (PCoA), Linear Discriminant Analysis (LDA), and Partial Least Squares Discriminant Analysis (PLS-DA) were tested, while normalization was carried out with CLR, ALR, and ILR transformations. A total of 7 machine learning algorithms was applied, 3 were linear models (logistic regression (LR), ridge regression (Ridge), support vector machine (SVM) with linear kernel), 3 were tree-based ensemble methods (random forest (RF), extra trees (ET), extreme gradient boosting (XGB)), and one was a neural network based on deep learning (multi-layer perceptron (MLP)). To enhance overall accuracy and mitigate both error and overfitting, a voting classifier (Ensemble) was implemented, consisting of an ensemble learning strategy that aggregates the outputs of the seven base learners through majority (hard) voting. The algorithms were retrieved from *scikit-learn* [26] and run on Python 3.13.2.

## Results

### Dataset composition and metagenomic profiling

The dataset of 569 fecal sample metagenomes from oncologic patients treated with different immunotherapeutic approaches was analyzed to explore the influence of microbiome composition on the clinical outcome of immunotherapy and to identify enterotype-specific biomarkers. The whole dataset (WD) included 262 R (46.0%) and 307 NR (54.0%), distributed as 292 MM (54.1% R), 239 NSCLC (37.5% R), and 38 RCC (36.8% R).

In the dataset, 4630 microbial taxa, 4602 *Bacteria* and 28 *Archaea*, were found*.* All indices of alpha diversity (Shannon index, Chao1 index, and Pielou’s evenness) showed comparable levels of species richness and evenness in R and NR groups. Similarly, no substantial differences in alpha diversity were observed among the different cancer types (Suppl. Figure 1).

**Fig. 1:**
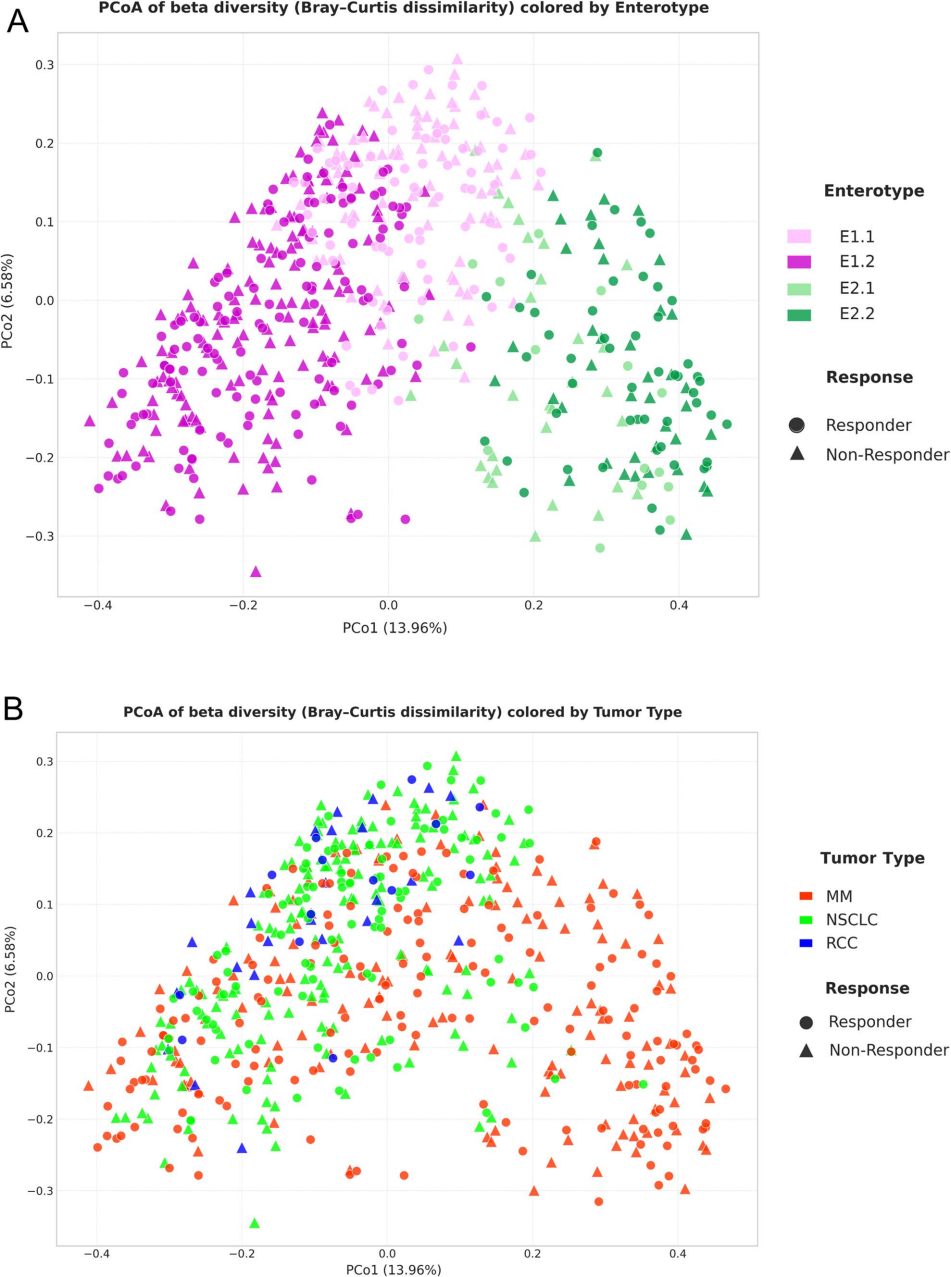
PCoA plot of beta diversity according to Bray–Curtis dissimilarity, displaying enterotypes (panel A) and tumor types (panel B). Subjects are shaped according to response (circle, R; triangle, NR)

Beta diversity of R and NR was assessed according to the Bray–Curtis dissimilarity index and analyzed with PCoA. In the plot of PCoA (Fig. 1) reporting the two most informative dimensions, the R and NR samples did not cluster separately and were generally overlapping. However, the bacterial composition of the two groups was different according to PERMANOVA (*p* = 0.043).

## Enterotypes

Metagenomes were clustered to identify groups with similar composition (enterotypes) utilizing the PAM algorithm. The algorithm was applied to the Bray–Curtis distance matrix of the 569 samples and generated the enterotypes E1 and E2. Each enterotype was further divided in the subgroups E1.1, E1.2, E2.1, and E2.2 utilizing the same clustering approach (Fig. 1).

**Fig. 2:**
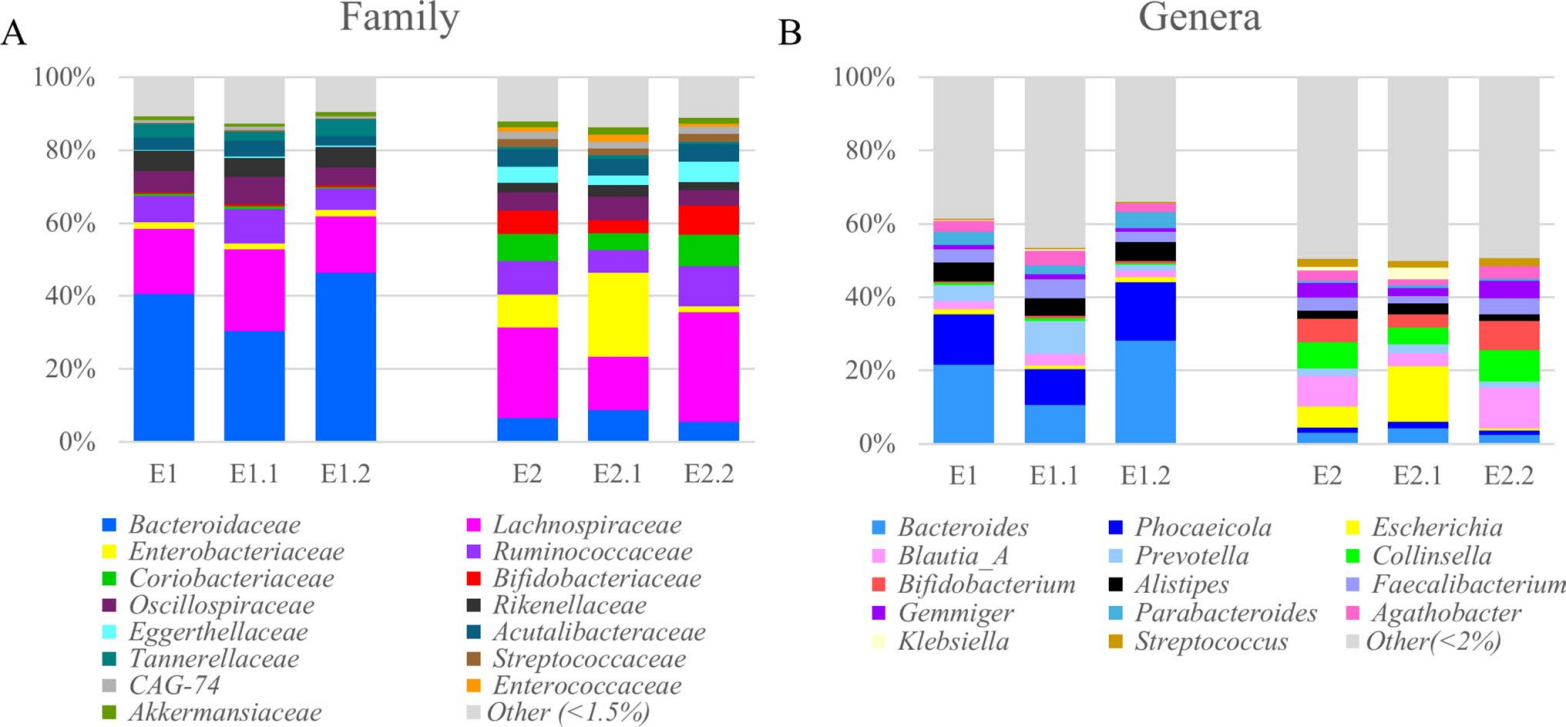
Mean microbiome composition at family (**A**) and genus (**B**) level of the enterotypes

The taxa belonging to family *Bacteroidaceae*, as well as the families *Rikenellaceae* and *Tannerellaceae* were the strongest drivers of difference of E1 compared with E2 (Fig. 2). E2 was characterized by higher abundance of *Lachnospiraceae, Enterobacteriaceae, Coriobacteriaceae, Bifidobacteriaceae*, and *Eggerthellaceae*. *Lactobacillales*, and in particular the families *Enterococcaceae* and *Streptococcaceae,* were also enriched in E2. At genus level, *Bacteroides, Phocaeicola, Prevotella*, and *Alistipes* were more abundant in E1, while *Escherichia, Blautia A, Collinsella, Bifidobacterium, Gemmiger*, and *Streptococcus* were more represented in E2.

Comparison between E1.1 and E1.2 revealed that the families *Lachnospiraceae, Ruminococcaceae, Oscillospiraceae*, and *Acutalibacteraceae* were more abundant in E1.1, while E1.2 was enriched with *Bacteroidaceae*. At genus level, E1.1 presented higher abundance of *Prevotella*, *Agathobacter*, and *Faecalibacterium*.

E2.1 was featured by a very high abundance of *Enterobacteriaceae* and by higher levels of *Oscillospiraceae* and *Enterococcaceae*, compared to E2.2. Conversely, the families *Lachnospiraceae, Ruminococcaceae, Coriobacteriaceae, Bifidobacteriaceae*, and *Eggerthellaceae* showed higher abundance in E2.2. At genus level, a high load of *Escherichia, Klebsiella*, and *Citrobacter* characterized E2.1, whereas *Blautia A, Collinsella, Bifidobacterium, Faecalibacterium*, and *Gemmiger* were more abundant in E2.2.

## Responders rates by enterotype

**Fig. 3:**
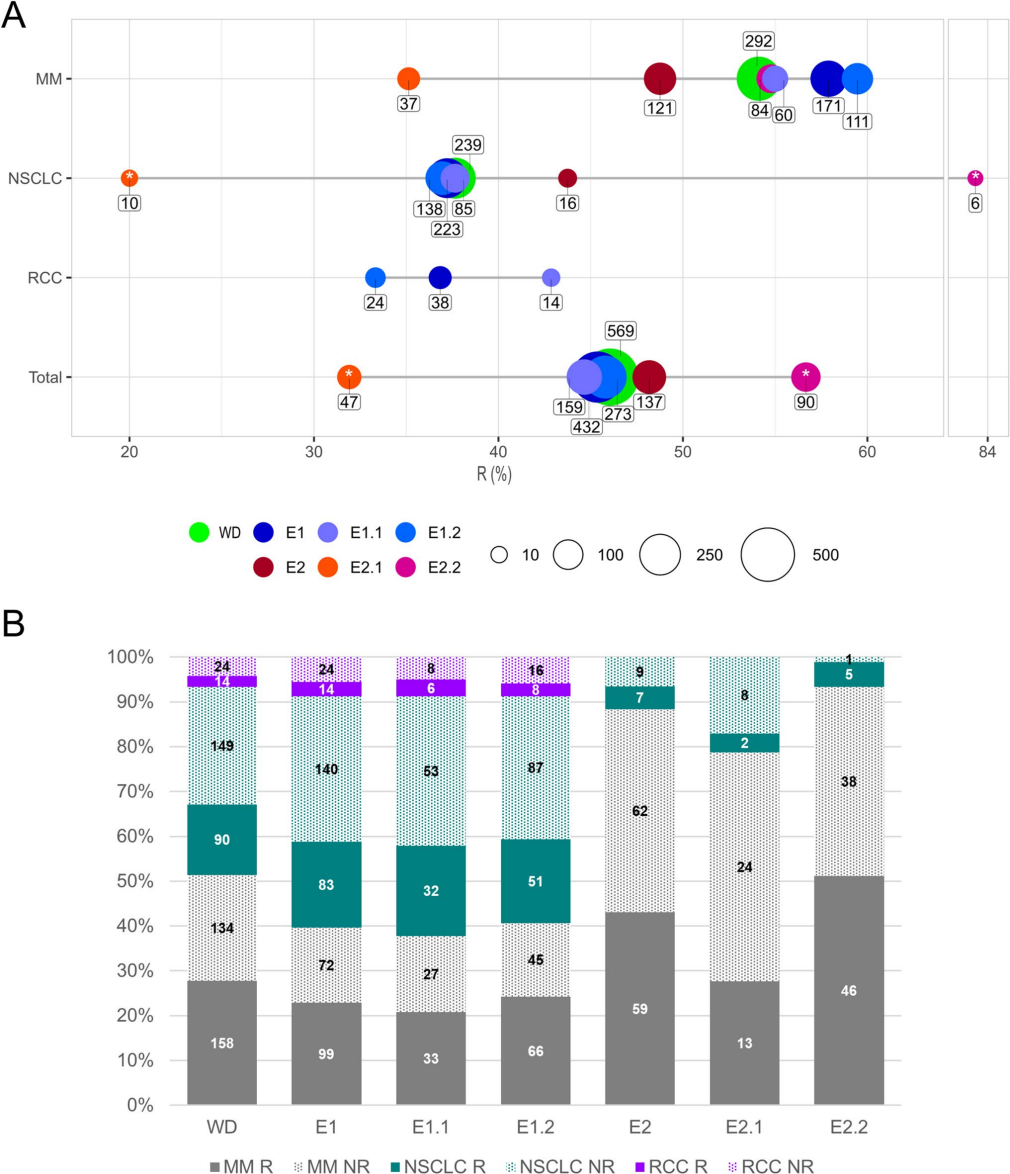
Response rate and tumor-type composition across enterotypes. **A** Dumbbell representation of the response rate (%) as a function of enterotypes and tumors. Dots correspond to the whole dataset (green) or enterotypes. The numerosity of each group (R + NR) is proportional to the dot size and is reported in labels. Stars indicate groups with a statistically significant difference in response rate (*p* < 0.05) compared with the parent group (Chi-squared and Fisher’s exact test, for WD and NSCLC, respectively). **B** Tumor-type composition of the whole dataset and each enterotype/subcluster, reported as absolute numbers and percentages of MM, NSCLC, and RCC samples

To address the potential confounding effect of tumor type on enterotype assignment, we explicitly quantified the distribution of cancer types across the whole dataset and each enterotype/subcluster, and we incorporated these data into Fig. 3 and Suppl. Table 2. Tumor-type composition was uneven across enterotypes. E1 comprised the majority of samples (432 out of 569; 196 R, 45.4% and 236 NR, 54.6%) and included all three tumor types: 171 MM cases (39.6%), 223 NSCLC cases (51.6%), and 38 RCC cases (8.8%). By contrast, E2 included 137 samples (66 R, 48.2%, and 71 NR, 51.8%) and was strongly enriched for MM, which accounted for 121 of 137 samples (88.3%), whereas NSCLC accounted for 16 samples (11.7%) and RCC was absent. E1.1 and E1.2 retained a mixed tumor composition similar to parent E1, whereas E2.1 and E2.2 remained MM-enriched: MM accounted for 37/47 samples (78.7%) and 84/90 samples (93.3%), respectively, NSCLC for 10/47 (21.3%) and 6/90 (6.7%), respectively. These data indicate that enterotype-response analyses, particularly those involving E2 and its subclusters, cannot be interpreted independently of tumor-type composition. Therefore, tumor-stratified comparisons are presented as descriptive and hypothesis-generating.

Within this descriptive framework, distinct response patterns were observed across enterotypes and tumor types. Taking into account cumulatively all cancer types, the response rates of E1, E1.1, and E1.2 were similar, suggesting no evident difference between the two E1 subclusters. By contrast, the response rate differed between E2.1 and E2.2 (Chi-squared test *p* = 0.010), with E2.1 showing a lower proportion of responders than E2.2 (31.9% and 56.7%, respectively). Given the strong MM enrichment and absence of RCC in E2, this difference should be interpreted as an enterotype-associated pattern observed within an unbalanced tumor-type context, rather than as evidence of an enterotype effect independent of tumor type.

All the RCC samples were assigned to E1, and a marked difference in response rate was observed between E1.1 (42.8%) and E1.2 (33.3%), albeit not significant. For NSCLC, E1.1 and E1.2 exhibited similar proportions of responders, whereas E2.1 and E2.2 showed markedly different proportions (Fisher’s exact test p = 0.035), with 5 out of 6 NSCLC patients in E2.2 responding to ICI therapy, against 2 out of 8 in E2.1. The E2.2 subgroup also showed a higher, but not significant, proportion of responders in MM patients (35.1% vs. 54.7% for E2.1 and E2.2, respectively).

## Identification of response-related biomarkers

For the whole dataset and for each enterotype, LEfSe was applied to discover distinctive features of response. Species identified as a biomarker in the whole dataset (WD) and/or in at least an enterotype are reported in Suppl. Figure 2.

A total of 166 species presented positive differential abundance in the responders of WD and/or in at least one enterotype, generating 247 enterotype-biomarker associations (Suppl. Figure 2). Biomarkers were unevenly distributed across enterotypes, reflecting distinct but partially overlapping microbial configurations associated with response. Despite this heterogeneity, several taxonomic groups emerged recurrently across multiple enterotypes, suggesting a conserved association with favorable immunotherapy outcome.

The genus *Collinsella* (*Coriobacteriaceae*) emerged as the main biomarker of positive response, especially in E2.1, where it accounted for 44 of the 61 response biomarkers. Fourteen responder-associated biomarkers were assigned to the genus *Bacteroides*, especially within enterotype E1 and its subgroups. *Longicatena caecimuris* (*Erysipelothricaceae*) were consistent markers of response in E1 and its subgroups.

Thirteen species belonging to the genus *Blautia* and *Blautia A* seemed robust responder-associated biomarkers, being identified across the whole dataset as well as in multiple enterotypes, including E1, E1.1, E1.2, and E2. Other *Lachnospirales*, such as *Anaerobutyricum, Anaerostipes, Clostridium AP scindens, Dorea, Eisenbergiella massiliensis, Eubacterium G ventriosum, Fusicatenibacter, Mediterraneibacter,* and *Ruminococcus A* were associated to response.

The *Monoglobales Monoglobus pectinilyticus* was biomarker of response in E1 and subgroups. Within *Oscillospirales*, the taxa consistently more abundant in R groups included *Anaeromassibacillus*, *Dysosmobacter welbionis*, and *Evtepia gabavorous*. Other taxa ascribed to *Peptostreptococcales* (*Romboutsia*) and *Acidaminococcales* (*Acidaminococcus intestinis*) were positively associated to the response, as well as different species of *Akkermansia*, including *A. muciniphila*.

Other taxa putatively relevant for response belonged to unassigned species, reconstructed from MAGs, i.e., the *Lachnospirales* clades (CAG127, CAG317, UBA11774, UBA7106, UMGS1375).

## Assessment of the consistency of responder-associated biomarkers

For the Modena Oncology Cohort, which included only 19 patients with exceptional and durable response to ICI, we performed an exploratory concordance analysis to determine whether the abundance of selected enterotype-specific species identified as responder-associated in the discovery dataset showed a responder-like distribution in an independent cohort of exceptional responders. These new samples included 9 MM, 7 NSCLC, and 3 RCC. After assigning each MOC sample to the closest enterotype/subcluster based on Bray–Curtis distance to the discovery medoids, the abundance of each selected species was compared with the responder and non-responder abundance observed in the corresponding discovery enterotype.

**Fig. 4:**
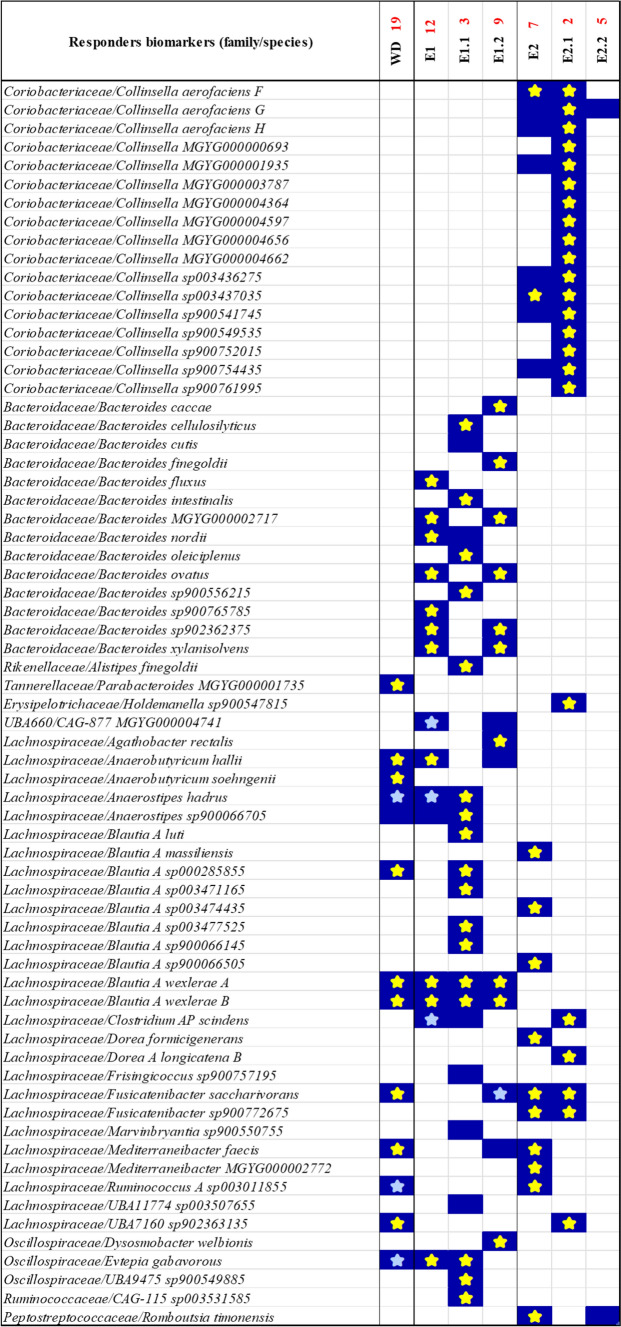
Bacterial biomarkers identified by LEfSe as presenting positive differential abundance in responders of the whole dataset (WD) and/or in at least one enterotype. Blue squares indicate statistical significance (LDA > 2, *p* < 0.05) within the whole dataset or a specific enterotype. Stars indicate biomarkers showing responder-like abundance patterns in > 80% (yellow) or > 75% (light blue) of the Modena Oncology Cohort (MOC) samples assigned to the corresponding enterotype in the exploratory concordance assessment

A species was considered concordant with the responder-associated pattern when most MOC samples assigned to that enterotype fell within the discovery responder-like abundance distribution. Because the number of MOC samples assigned to some enterotypes was small, these results were interpreted descriptively and not as evidence of predictive performance. In particular, for enterotypes 1.2, 2.1, and 2.2, for which only 2 to 5 assessment samples were available, the observed abundance patterns were reported descriptively. The ESS was consistent with the responder-associated pattern when all abundances in the available MOC samples matched the R distribution. Figure 4 presents all the ESS supported in at least one enterotype.

With a few exceptions, most ESS of *Anaerobutyricum, Anaerostipes, Bacteroides, Blautia A, Dorea, Fusicatenibacter*, and *Mediterraneibacter* showed concordant abundance patterns in at least one enterotype. For these species, other ESS-enterotype associations that did not reach the 80% threshold remained at 75%.

All 14 *Bacteroides* species predicted as biomarkers were supported in the preliminary assessment, and the only unsupported taxon, *B. cutis*, was still concordant in more than 66% of the samples. Fourteen *Collinsella* ESS associated with E2.1 were confirmed, albeit on data from only two patients, with *C. aerofaciens F* and *Collinsella sp003437035* also showing concordance in E2. All 17 predicted associations between *Blautia A* species and enterotypes were supported by the MOC samples. Species assigned to the genus *Blautia A* represented the most consistent responder-associated signal. Both *Blautia A wexlerae A* and *Blautia A wexlerae B* showed concordant abundance patterns across the entire dataset (WD), as well as within E1, E1.1, and E1.2. Five additional *Blautia A* species were confirmed in E1.1, while three distinct *Blautia A* species were consistently supported in E2.

Additional taxa, such as *Agathobacter rectalis*, *Alistipes finegoldii*, *Clostridium AP scindens*, *Dysosmobacter welbionis*, *Evtepia gabavorous*, and *Romboutsia tomonensis*, were likewise confirmed in at least one enterotype. *Fusicatenibacter saccharivorans* and *F. sp900772675* showed concordant abundance patterns within E2 and E2.1, with *F. saccharivorans* additionally supported across the full dataset WD. Likewise, *Mediterraneibacter* spp. were consistently confirmed across the dataset and within E2.

Except for E2.2, for which no ESS reached the confirmation threshold, the proportion of supported ESS in the different enterotypes ranged between 30 and 43%. Despite the small sample size per enterotype (ranging from 2 to 12), these findings indicate that some responder-associated biomarkers have higher potential, being confirmed in different enterotypes.

## Machine learning classification

Machine learning methods were applied to the whole dataset to perform classification tasks aimed at distinguishing responders (CR + PR) from non-responders (PD + D) to immunotherapy treatment. The classification utilized 4630 features at species level and 1128 at genus level. The preprocessing strategies based on data reduction and normalization (PCA, KPCA, t-SNE, PCoA, LDA, and PLS-DA) did not clearly identify one method as generally more effective than another.

Using the species dataset, seven models (LR, Ridge, SVM, RF, ET, XGB, and MLP) were tested individually and in an ensemble voting classifier. Individually, the models exhibited limited predictive accuracy, ranging between 0.518 and 0.614 (Table 2). The ensemble approach achieved the most stable performance, yielding the highest overall accuracy of 0.640. Interestingly, when focusing on the CR subgroup of the WD test set, consisting of patients who exhibited a complete response to immunotherapy, the ensemble classifier reached an accuracy of 0.800. In the MOC, which exclusively comprised highly responsive patients, 84.2% of samples were assigned to the responder class, providing concordance in a responder-only cohort.

**Table 2:** Classification accuracy of machine learning models using species- or genus-level taxonomy to distinguish responders (CR + PR) from non-responders (PD + D) to immunotherapy

Feature set	Split	LR	Ridge	SVM	MLP	RF	ExtraT	XGB	Ensemble
Species-level taxonomy	Test set (held-out; 114)	0.614	0.588	0.579	0.588	0.526	0.570	0.518	0.640
	Test set (only CR; 15)	0.533	0.733	0.733	0.933	0.600	0.867	0.667	0.800
	MOC (CR, 19)	0.526	0.579	1.000	1.000	0.526	0.895	0.632	0.842
Genus collapsed	Test set (held-out; 114)	0.553	0.579	0.561	0.561	0.561	0.544	0.553	0.579
	Test set (only CR, 15)	0.750	1.000	1.000	0.667	0.583	0.750	0.667	0.833
	MOC (CR, 19)	0.947	1.000	0.789	0.526	0.842	0.632	0.789	0.947

Collapsing the taxonomic profiles at the genus level further increased the proportion of MOC samples assigned to the responder class to 0.947. Conversely, on the general held-out test set, the ensemble's accuracy decreased from 0.640 (species-level taxonomy) to 0.579 (genus-collapsed taxonomy), highlighting a trade-off: taxonomic aggregation may strengthen intra-cohort concordance while reducing fine-grained discriminatory power across more heterogeneous populations.

## Discussion

Several studies highlighted the role of gut microbiota in the response to cancer immunotherapy across various cancer types, pinpointing putative biomarkers related to positive response to ICI [7, 11, 29–31]. The identification of specific signatures associated with favorable outcomes of ICI treatment remains inconsistent among studies, probably also due to limited study size, heterogeneous treatment strategies, and technical variability across cohorts. This study analyzed a large microbiome dataset (569 samples) to explore whether enterotype-based stratification could facilitate the detection of response-associated biomarkers in a heterogeneous multi-cohort setting.

We assumed that the variety of microbiome profiles could hinder the identification of common signatures useful to predict the response to checkpoint blockade therapies. For this reason, the 569 metagenomes were clustered into enterotypes based on similar composition, regardless of tumor type and immunotherapeutic intervention, with the aim of detecting specific biomarkers of response within groups of similar microbiomes. Partitioning the samples using the PAM algorithm resulted in two main enterotypes, E1 and E2, which were further divided into two subclusters. We are aware that clustering can be performed using different approaches. In this proof-of-concept study, enterotype separation was intended to highlight the specificity of response biomarkers within groups of similar microbiomes and to explore whether high inter-individual variability may impede the identification of differential markers.

The integrated dataset combines public cohorts generated across different hospitals and experimental procedures, so residual batch effects and cohort-specific signals may have influenced both enterotype structure and biomarker discovery. The standardized downstream bioinformatic workflow reduced but could not fully remove heterogeneity arising from differences in sample collection, storage, DNA extraction, library preparation, sequencing depth, and study/hospital provenance.

Some enterotypes, particularly E2.1 and E2.2 subclusters, displayed different responder proportions. However, the interpretation of these differences is constrained by the uneven tumor-type distribution across enterotypes: E2 and its subclusters were strongly enriched for MM and did not include RCC samples. Therefore, the observed responder proportions may reflect a combination of microbiome structure, tumor type, cohort/study effects, and response status. Within these limitations, the difference between E2.1 and E2.2 prompted us to search for microbial signatures separating the two subclusters: *Escherichia coli D* and its higher taxonomic levels up to the phylum *Proteobacteria* were the main drivers of difference enriched in E2.1.

*E. coli* produces lipopolysaccharides (LPS) that promote inflammatory signaling and contribute to barrier disruption and intestinal inflammation. The abundance of *E. coli* and *Enterobacteriaceae* could be related to the lower proportion of responders, since these taxa are generally associated with unfavorable clinical outcomes and inflammatory conditions, often contrasting with beneficial commensals. Accordingly, in NSCLC patients, species belonging to *Gammaproteobacteria* (including *E. coli*) were dominant in patients with overall survival < 12 months [11] and, in NSCLC non-responder patients treated with anti-PD-1/PD-L1, the genus *Escherichia–Shigella* resulted more abundant than in responders [32]. Microbiome composition may contribute to the success or failure of immunotherapy and targeted strategies aimed at microbiota can be identified to shift its composition from a negative to a positive enterotype. For example, modulatory approaches designed to reduce *E. coli* and *Enterobacteriaceae* in E2.1 patients may shift the microbiome toward E2.2, thereby increasing the likelihood of therapeutic success.

The 166 ESS identified were subjected to an exploratory concordance assessment in a cohort of 19 oncologic patients with exceptional and durable response to ICI therapy. Because this cohort included only exceptional responders, it cannot provide predictive validation or estimate discriminatory performance against non-responders. A major limitation of the MOC assessment is the absence of local non-responder controls. Therefore, this cohort cannot establish whether the selected taxa discriminate responders from non-responders in an independent real-world population. Rather, it provides only preliminary evidence that some taxa identified as responder-associated in the discovery dataset can also be detected with responder-like abundance patterns in an independent group of exceptional responders.

The MOC cohort should be interpreted as an exploratory enrichment of patients with exceptionally favorable and durable benefit from ICI, rather than as a cohort selected for early response per se. Accrual of such cases is inherently difficult across tumor types, because deep and/or long-lasting responses represent only a minority of ICI-treated patients under stringent clinical definitions. Within this context, our single-center assessment provided a preliminary exploratory step aimed at determining whether selected enterotype-associated signals could be observed again in an independent, clinically enriched cohort. Robust validation will require a larger multicenter study including both responders and non-responders, with harmonized eligibility criteria, standardized sample collection and processing, and pre-specified tumor-stratified analyses to adequately assess reproducibility, discrimination, and generalizability across clinical settings. Despite the limited sample size within each enterotype, the MOC analysis highlighted concordant responder-like abundance patterns for selected biomarkers within specific enterotypes. In particular, *Blautia A*, *Collinsella*, and a subset of *Bacteroides* species emerged as recurrent signals warranting further validation.

In patients assigned to enterotype E2.1, *Collinsella* abundance was associated with responder-enriched microbiome configurations. Numerous *Collinsella* species were enriched in responders within E2.1, the subgroup with the lowest descriptive response rate, suggesting a potential counterbalancing association within a microbiota otherwise enriched in *Enterobacteriaceae*. In this context, *Collinsella* may represent a taxon of interest for further mechanistic investigations.

*Collinsella aerofaciens* has already emerged as a gut microbial taxon of interest in the context of cancer immunotherapy [8, 33, 34]. Furthermore, fecal microbiota transplantation (FMT) in germ-free mice with responder microbiota enriched, among other taxa, *C. aerofaciens*, and improved the success of ICI enhancing T cell activation and promoting pro-inflammatory environments [35]. This is consistent with its pro-inflammatory activity linked with liver inflammation and with pathophysiology of non-alcoholic fatty liver disease, rheumatoid arthritis, and spondylarthritis [36–38]. Beyond its reported enrichment in responders to ICI, *C. aerofaciens* produces a pH-responsive lipid immunogen capable of activating innate immune signaling pathways, including TLR2-dependent responses [39]. This immunostimulatory capacity provides a plausible mechanistic link between *Collinsella* abundance and enhanced antitumor immunity. It may contribute to shaping a pro-inflammatory yet immunotherapy-permissive tumor microenvironment, thereby supporting durable responses to immune checkpoint blockade.

Some species of *Bacteroides* stood out as major biomarkers of response of E1, E1.1 and E1.2. Since the genus *Bacteroides* emerges as a biomarker of response only in E1, it may warrant further investigation whether these species could interact with the host through specific molecular mechanisms that promote responsiveness to immunotherapy. *B. ovatus* is strongly associated with host health [40] and emerges as a promising next-generation probiotic due to its capacity to modulate host metabolism and influence disease-related physiological processes. Like the other *Bacteroides* spp., it participates in the catabolism of complex carbohydrates and produces short-chain fatty acids (SCFAs), which support gut barrier integrity and beneficial metabolic signaling. It is also involved in bile acid metabolism, contributing to improvements in glucose tolerance and insulin sensitivity in preclinical studies. Variations in its abundance have been correlated with clinical states such as inflammatory bowel disease and metabolic disorders, suggesting a role in disease modulation.

Antitumor effects of CTLA-4 blockade depend on distinct *Bacteroides* species that enhance CTLA-4 inhibitor efficacy through an interleukin-12 dependent T helper 1 (TH1) immune response [41]. In a humanized microbiome mouse model of glioma, gut microbial composition was shown to critically influence the efficacy of anti-PD-1 immunotherapy [42]. In this preclinical setting, *B. cellulosilyticus* was consistently enriched in responder microbiomes compared with non-responders. Its higher abundance was associated with improved survival following immune checkpoint blockade. Moreover, previous evidence suggests that *B. caccae* may be associated with clinical response to immune checkpoint inhibitor (ICI) therapy [6, 10, 43]. In a large metagenomic study of NSCLC patients receiving immunotherapy, *B. caccae* emerged as a species significantly enriched in responders and contributed to predictive microbiome-based classifiers of treatment efficacy. These findings extend earlier clinical observations in melanoma cohorts, where baseline enrichment of *B. caccae* was reported in patients achieving favorable outcomes under checkpoint blockade. Accumulating evidence from microbiome profiling studies suggests that also *B. xylanisolvens* may be associated with responsiveness to immune checkpoint inhibitor therapy [44, 45]. In clinical cohorts of melanoma patients treated with anti-PD-1 or anti-CTLA-4 antibodies, *B. xylanisolvens* has been reported among bacterial species enriched in responders compared with non-responders. Together, available data and our results indicate that selected species of *Bacteroides*, i.e., *B. ovatus, B. cellulosilyticus, B. xylanisolvens,* and *B. caccae,* may contribute to a microbiome configuration permissive to effective antitumor immune responses shaping systemic antitumor immunity and representing a microbial biomarker or modulator of response to ICI.

A number of relevant studies associated *Akkermansia* to ICI response [7, 11, 46, 47]. For instance, Routy et al. [7] provided both clinical and mechanistic evidence linking *A. muciniphila* to improved efficacy of PD-1-based immunotherapy, supporting a causal contribution of this species to immunotherapy response. Furthermore, the large metagenomic NSCLC study by Derosa et al. [11] demonstrated that baseline fecal abundance of *A. muciniphila* was significantly associated with higher objective response rates and prolonged overall survival under PD-1/PD-L1 blockade, independent of key clinical confounders, providing strong clinical evidence that *Akkermansia* enrichment at treatment initiation correlates with improved immunotherapy efficacy. Consistently, the analysis of the whole dataset and of some enterotypes identified *Akkermansia* spp. as potential biomarkers, but the preliminary assessment with the 19 MOC patients did not corroborate these results. Likely, *Akkermansia* was an enterotype-specific biomarker less robust than *Blautia A*, *Collinsella*, and *Bacteroides*, and the low numerosity of the MOC was insufficient for validation.

Some studies have associated *Faecalibacterium*, particularly *Faecalibacterium prausnitzii*, with improved outcomes during immune checkpoint inhibitor (ICI) therapy. In metastatic melanoma treated with anti–CTLA-4, enrichment in *Faecalibacterium* at baseline was linked to longer progression-free and overall survival [48]. Subsequent metagenomic analyses in anti–PD-1/PD-L1–treated cohorts, including NSCLC and multi-cancer populations, identified higher baseline *F. prausnitzii* abundance in responders compared to non-responders [49, 50]. The absence of *F. prausnitzii* as a biomarker in our analysis, both globally and within enterotypes, likely reflects the context-dependent and non-universal nature of its reported associations. The role of *F. prausnitzii* may be more indicative of general gut eubiosis than of ICI-specific immunomodulation. In the large, heterogeneous pan-cancer dataset, its high baseline prevalence, functional redundancy within enterotypes, and differences in analytical pipelines and clinical endpoints may further limit its discriminatory power.

In parallel with enterotype- and biomarker-based analyses, we evaluated the potential of artificial intelligence approaches applied to metagenomic profiles to predict responder and non-responder status to immunotherapy. Machine learning models trained on the full dataset achieved moderate performance, with a maximum accuracy of 0.64 using an ensemble classifier. Notably, predictive accuracy increased when analyses were restricted to clinically more homogeneous subsets, such as patients with complete response and the MOC, reaching up to 0.80–0.84 and 0.95 when using genus-level profiles. These findings indicate that AI-based models do not identify universal microbial signatures of response, but rather capture complex compositional patterns associated with strong therapeutic benefit.

The pronounced inter-individual microbiome heterogeneity, also reflected by beta-diversity analyses, suggests that predictive performance may benefit from prior stratification of patients into microbiome-defined groups, such as enterotypes. However, broader confirmation will require larger multicenter cohorts, prospective designs, and balanced independent validation sets including both responders and non-responders.

As a whole, these preliminary findings may provide the rationale for a more structured and adequately powered multicenter validation study including both responders and non-responders. A larger design of this kind would enable a more robust evaluation of the reproducibility, discriminatory ability, and clinical relevance of enterotype-associated microbial biomarkers across heterogeneous patient populations. More broadly, such an approach could accelerate the generation of new evidence on microbiome features linked to ICI benefit and lay the groundwork for personalized microbiome-modulating strategies, including nutraceutical, probiotic, and postbiotic interventions, with the long-term aim of improving immunotherapy outcomes.

## Supplementary Information

Below is the link to the electronic supplementary material.

Supplementary file1 (XLSX 27 KB)

Supplementary file2 (PDF 2463 KB)

Supplementary file3 (DOCX 99 KB)

## Data Availability

Accession numbers of publicly available metagenomes utilized in this work are reported in Table 2 and Suppl. Spreadsheet 1. The raw sequences of the 19 MOC responders that were sequenced for this work are available on NCBI repository on Bioproject PRJNA1421997.
