# Supplementary material for: Enterotype-specific microbial biomarkers of immune checkpoint inhibitor response revealed by large-scale integrated metagenomic analysis
Source: Cancer Immunol Immunother. 2026 May 26;75(8):193. doi: 10.1007/s00262-026-04432-w (PMC13388844; doi:10.1007/s00262-026-04432-w)

Supplementary Figure 2: Bacterial biomarkers identified by LeFSe as presenting positive differential abundance in the responders of the whole dataset (WD) and/or in at least one enterotype. Blue squares indicate statistical significance (LDA > 2, p < 0.05) within the whole dataset or a specific enterotype. Stars indicate that the biomarkers were confirmed in the Modena Oncology Cohort (MOC). In red it is reported the number of samples belonging to the MOC attributed to each enterotype.

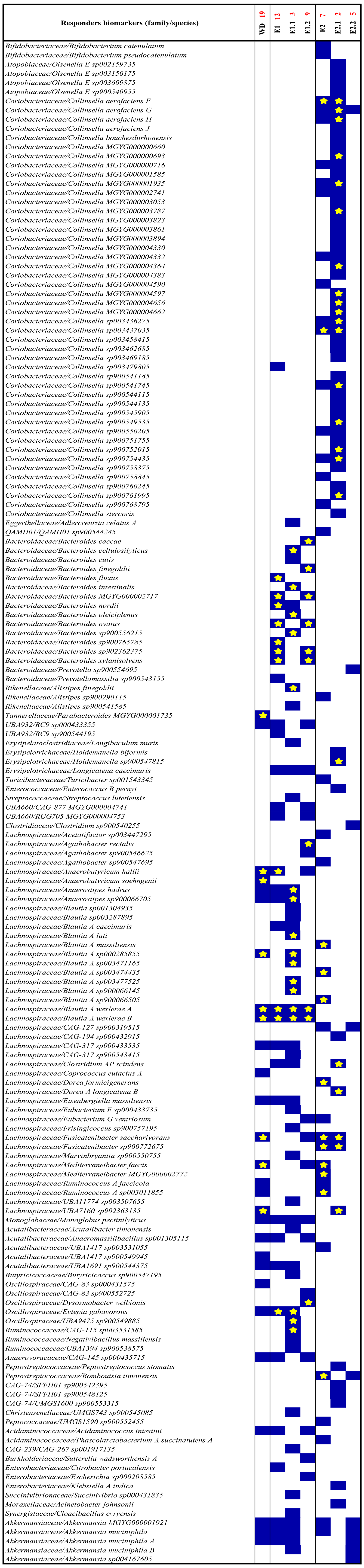

Supplement: Supplementary file 2 — Supplementary file2 (PDF 2463 KB) [file 262_2026_4432_MOESM2_ESM.pdf]
