# Supplementary material for: Enterotype-specific microbial biomarkers of immune checkpoint inhibitor response revealed by large-scale integrated metagenomic analysis
Source: Cancer Immunol Immunother. 2026 May 26;75(8):193. doi: 10.1007/s00262-026-04432-w (PMC13388844; doi:10.1007/s00262-026-04432-w)
Supplement: Supplementary file 3 — Supplementary file3 (DOCX 99 KB) [file 262_2026_4432_MOESM3_ESM.docx]

**Enterotype-Specific Microbial Biomarkers of Immune Checkpoint Inhibitor Response Revealed by Large-Scale Integrated Metagenomic Analysis**

Running title: Enterotype-Specific Microbial Biomarkers

**Francesco Candeliere^1^, Enrico Busi^1^, Sara Cerri**^2^**, Laura Sola^1^, Matteo Lombardi^3^, Stefano Greco^4,5^, Sara Pedroni^1^, Alberto Amaretti^1,6^, Stefano Raimondi^1,6^, Chiara Chiavelli^4^, Maria Giuseppa Vitale^5^, Federica Bertolini^5^, Roberta Depenni^5^, Giorgia Franchini^3^, Massimo Dominici^4,5*^ & Maddalena Rossi^1,6*^**

*^1^* *Department of Life Sciences, University of Modena and Reggio Emilia, Modena, 41125, Italy.*

*^2^ Operative Unit of Oncology, Department of Oncology, ASST Cremona – Hospital of Cremona, Cremona, 26100, Italy*

*^3^Department of Physics, Informatics and Mathematics, University of Modena and Reggio Emilia.*

*^4^ Division of Oncology, Department of Medical and Surgical Sciences for Children & Adults, University of Modena and Reggio Emilia, Modena*

*^5^ Division of Oncology, Department of Oncology and Hematology, University of Modena & Reggio Emilia and University Hospital of Modena, Modena, Italy*

*^6^Biogest-Siteia, University of Modena and Reggio Emilia, Modena*

^*^  Corresponding authors

Francesco Candeliere and Enrico Busi contributed equally to this study.

**Correspondence to:**

Prof. Maddalena Rossi, Department of Life Sciences, University of Modena and Reggio Emilia, Via Campi 103, Modena, 41125, Italy. E-mail: maddalena.rossi@unimore.it ORCID: https://orcid.org/0000-0002-5342-3950

Prof. Massimo Dominici, Division of Oncology, Department of Medical and Surgical Sciences for Children & Adults, University of Modena and Reggio Emilia, Largo del Pozzo 71, Modena, 41125, Italy. E-mail: massimo.dominici@unimore.it ORDIC: https://orcid.org/0000-0002-4007-1503

**Supplementary material**


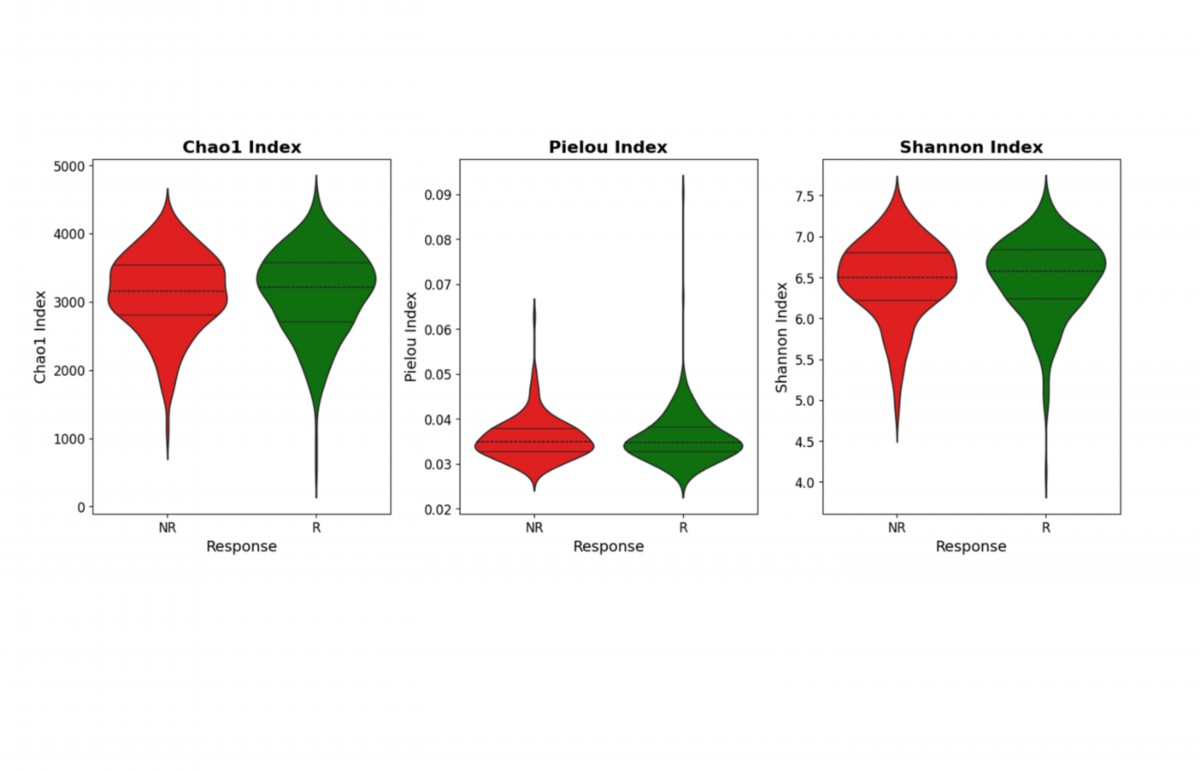


*Supplementary Figure 1: Alpha diversity of the samples calculated according to Chao1, Pielou and Shannon indices. NR samples are reported in red, while R in green. No statistical significance was found (Kruskal-Wallis test, p > 0.05).*

*Supplementary Table 1: Characteristics of the Modena oncology cohort (MOC) patients. Age, gender (M=Male; F=Female), cancer type (NSCLC=Non-Small Cell Lung Cancer; MM=Malignant Melanoma; RCC=Renal Cell Carcinoma), treatment (*Combination therapy for 4 cycles, then maintenance phase with only anti-PD1) and best response (CR=Complete Response; PR=Partial Response) are reported. These patients presented an “extraordinary” response to ICI, defined as a CR or PR lasting for more than 2 years.*

| **Patient** | **Age (years)** | **Gender** | **Cancer type** | **Treatment** | **Best response** |
| --- | --- | --- | --- | --- | --- |
| 1 | 59 | M | RCC | Anti-PD1 + Anti-CTLA4* | CR |
| 2 | 75 | M | MM | AntiPD-1 | CR |
| 3 | 84 | F | NSCLC | AntiPD-1 | PR |
| 4 | 63 | M | NSCLC | AntiPD-1 | PR |
| 5 | 73 | M | MM | AntiPD-1 | PR |
| 6 | 75 | M | NSCLC | AntiPD-1 | PR |
| 7 | 71 | F | NSCLC | AntiPD-1 | CR |
| 8 | 65 | M | RCC | Anti-PD1 + Anti-CTLA4* | CR |
| 9 | 75 | M | RCC | AntiPD-1 | PR |
| 10 | 58 | M | MM | AntiPD-1 | CR |
| 11 | 75 | F | NSCLC | AntiPD-1 | CR |
| 12 | 75 | M | MM | AntiPD-1 | CR |
| 13 | 66 | F | NSCLC | AntiPD-1 | CR |
| 14 | 79 | F | MM | AntiPD-1 | CR |
| 15 | 73 | F | MM | AntiPD-1 | CR |
| 16 | 66 | F | MM | AntiPD-1 | CR |
| 17 | 79 | M | MM | AntiPD-1 | CR |
| 18 | 61 | M | MM | AntiPD-1 | CR |
| 19 | 74 | F | NSCLC | AntiPD-1 | PR |

*Supplementary Table 2: Distribution of samples in the whole dataset (WD), clusters and subcluster considering tumor type and response. In parentheses the percentage of samples’ number relative to the total number of samples in each group is reported.*

|  | **MM** | | | **NSCLC** | | | **RCC** | | | **Tot R (%)** | **Tot NR (%)** | **Tot samples** |
| --- | --- | --- | --- | --- | --- | --- | --- | --- | --- | --- | --- | --- |
|  | **R (%)** | **NR (%)** | **Tot (%)** | **R (%)** | **NR (%)** | **Tot (%)** | **R (%)** | **NR (%)** | **Tot (%)** |  |  |  |
| **WD** | 158 (27.8) | 134 (23.6) | 292 (51.3) | 90 (15.8) | 149 (26.2) | 239 (42) | 14 (2.5) | 24 (4.2) | 38 (6.7) | 262 (46) | 307 (54) | 569 |
| **E1** | 99 (22.9) | 72 (16.7) | 171 (39.6) | 83 (19.2) | 140 (32.4) | 223 (51.6) | 14 (3.2) | 24 (5.6) | 38 (8.8) | 196 (45.4) | 236 (54.6) | 432 |
| **E1.1** | 33 (20.8) | 27 (17) | 60 (37.7) | 32 (20.1) | 53 (33.3) | 85 (53.5) | 6 (3.8) | 8 (5) | 14 (8.8) | 71 (44.7) | 88 (55.3) | 159 |
| **E1.2** | 66 (24.2) | 45 (16.5) | 111 (40.7) | 51 (18.7) | 87 (31.9) | 138 (50.5) | 8 (2.9) | 16 (5.9) | 24 (8.8) | 125 (45.8) | 148 (54.2) | 273 |
| **E2** | 59 (43.1) | 62 (45.3) | 121 (88.3) | 7 (5.1) | 9 (6.6) | 16 (11.7) | 0 | 0 | 0 | 66 (48.2) | 71 (51.8) | 137 |
| **E2.1** | 13 (27.7) | 24 (51.1) | 37 (78.7) | 2 (4.3) | 8 (17) | 10 (21.3) | 0 | 0 | 0 | 15 (31.9) | 32 (68.1) | 47 |
| **E2.2** | 46 (51.1) | 38 (42.2) | 84 (93.3) | 5 (5.6) | 1 (1.1) | 6 (6.7) | 0 | 0 | 0 | 51 (56.7) | 39 (43.3) | 90 |
